# Development of Soil Compaction Analysis Software (SCAN) Integrating a Low Cost GPS Receiver and Compactometer

**DOI:** 10.3390/s120302351

**Published:** 2012-02-23

**Authors:** Jinsang Hwang, Hongsik Yun, Juhyong Kim, Yongcheol Suh, Sungnam Hong, Dongha Lee

**Affiliations:** 1 Department of Civil, Architectural & Environmental Engineering, Sungkyunkwan University, Suwon 440-746, Korea; E-Mails: gpsboy@skku.edu (J.H.); yoonhs@skku.edu (H.Y.); 2 Geotechnical Engineering Research Division, SOC Research Institute, Korea Institute of Construction Technology, Goyang 411-712, Korea; E-Mail: haitink@kict.re.kr; 3 Department of Spatial Information Engineering, Pukyong National University, Busan 608-737, Korea; E-Mail: suh@pknu.ac.kr

**Keywords:** GPS, compactometer, Kalman filter, spatial object modeling, intelligent compaction

## Abstract

A software for soil compaction analysis (SCAN) has been developed for evaluating the compaction states using the data from the GPS as well as a compactometer attached on the roller. The SCAN is distinguished from other previous software for intelligent compaction (IC) in that it can use the results from various types of GPS positioning methods, and it also has an optimal structure for remotely managing the large amounts of data gathered from numerous rollers. For this, several methods were developed: (1) improving the accuracy of low cost GPS receiver’s positioning results; (2) modeling the trajectory of a moving roller using a GPS receiver’s results and linking it with the data from the compactometer; and (3) extracting the information regarding the compaction states of the ground from the modeled trajectory, using spatial analysis methods. The SCAN was verified throughout various field compaction tests, and it has been confirmed that it can be a very effective tool in evaluating field compaction states.

## 1. Introduction

Soil compaction represents a significant portion of construction budgets, and is very important to the performance and stability of soil structures. However, the required compaction level is difficult to achieve due to the heterogeneity of earth materials, variations in equipment and operators, and difficulty in maintaining uniform lift thickness and moisture content [1]. Generally, static or vibrating rollers have been used for compaction work. In the case of a vibrating roller, it has constant frequency and amplitude of its vibration, so workers are only able to control compaction work by keeping the same speed of the roller and changing the number of driving times for the same path. This process often results in an unevenly compacted earthwork area in which a certain part of the area is sufficiently compacted and the remainder is over-compacted or insufficiently compacted [2].

A new compaction method called intelligent compaction (IC) has been used in recent years. This new method aims at providing a higher quality road or embankment by implementing a reliable quality and interactive assurance system from the outset of a construction [2]. IC rollers use either accelerometers and/or machine energy to calculate an index parameter related to modulus, stiffness, or bearing capacity. This information is then used by the roller’s control systems to determine whether to increase or decrease compaction energy by automatically adjusting the internal, mechanical parameters of the roller [3]. The main IC process operates on an application software for analyzing data from sensors on the IC roller, displaying the compaction results, and supporting a decision for quality assurance.

This research focuses on the application software for the soil compaction analysis which is part of the IC process. Today, available IC software supports only limited functionality: “BCM 05 Positioning” of Bomag, “ACEplus” of Ammann and “Compaction Viewer” of Caterpillar *etc.* [4]. These software support functions for analyzing compaction information from the sensors of a compaction roller by linking Global Positioning System (GPS) positioning results and helping workers access the sufficient compaction adequate for their own purposes. They can analyze compaction times, the relative or absolute modulus of compaction for a ground and display analysis results which are used for controlling the compaction roller.

Similar to the above software, the Soil Compaction ANalysis (SCAN) software is designed to be used as a part of the IC system for analyzing compaction data, but it has two advanced features. The first distinctive feature of SCAN is its function of utilizing various types of GPS receivers. Existing IC systems use high cost GPS equipment supporting Real Time Kinematic (RTK) positioning, of which the analysis software is largely dependent upon the positioning results from the GPS equipment, so it uses the results from the GPS equipment without any processing. These limitations lead to raising the price of the positioning part of the entire IC system. However, SCAN supports not only RTK positioning, but also Satellite Based Augmentation System (SBAS) positioning and Single Point Positioning (SPP). This makes it possible to adopt low cost GPS equipment in the IC process. For this, the Kalman Filter (KF) [5–7], which uses velocity calculated from a GPS receiver as a control variable, and other filters were developed to improve the accuracy of positioning result from low cost GPS receiver.

The second distinctive feature of SCAN is the adequate structure for not only monitoring many rollers in real time but the precise modeling of the roller’s trajectory. Most commercial IC software have been developed only for supporting a roller’s driver, but there is a need to monitor every roller in real time to more precisely control the quality of the compaction work. To meet this need, adequate spatial object models for modeling a roller’s trajectory has been developed, and optimal spatial indexing and spatial analysis methods have been implemented in SCAN.

In this paper, the developed methods for these distinctive features of SCAN are described in detail, and the results of a field test that was conducted to verify the performance of such methods are presented. Most of the papers on IC published thus far focus on the overall composition of IC or on the methodology of relating the sensed data with the soil compaction modulus. Anderegg *et al*. [8] presented the concept of continuous compaction control and illustrated methods of analyzing soil compaction data in conjunction with the GPS coordinates. Camargo *et al*. [9] showed an example of a case where GPS-related soil compaction data were displayed on ESRI’s ArcMap software, overlaid with aerial photographs and a vector map. Briaud and Seo, [2] presented a commercial application software related to IC, and presented their analysis results. It is difficult, however, to find a detailed description of methods for the processing, analysis, and displaying of GPS-related compaction data. This paper includes many software methods for these, making it distinct from the previous related researches, and can thus be used as a reference for the related research.

## 2. Application Environments for SCAN

### 2.1. IC System Configuration

**Figure 1.**
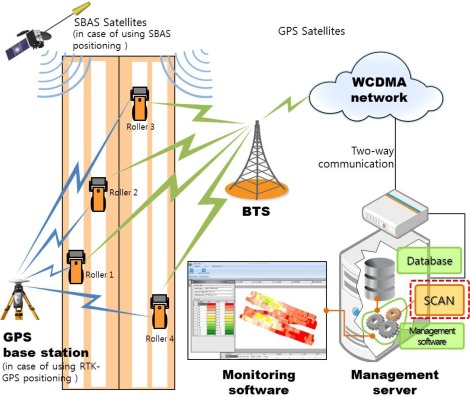
IC system configuration.

SCAN is designed to be used as a compaction data analysis module for an IC system that monitors many rollers simultaneously. Each monitored roller is equipped with GPS, a compactometer and wireless communication modems and sends compaction information to the management server in real time. Figure 1 shows the overall configuration of the IC system that uses SCAN.

Two types of satellites are used in the IC system. One is the GPS satellite which transmits signals for positioning and the other is the SBAS satellite which transmits correction messages for GPS positioning. There are two types of correction methods for the GPS positioning: the code-based differential GPS (DGPS) method and the carrier-phase based DGPS method [10]. In the case of using SBAS correction messages, the code-based DGPS is able to determine a position with an accuracy of about one-meter (rms) error [11,12]. The RTK positioning, which uses RTCM 2.3 or other messages from one or more ground GPS base stations, the carrier-phase based DGPS, enables a position to be determined with an accuracy of few centimeters (rms) error [11,12].

### 2.2. Equipment and Sensor Implementation

**Figure 2.**
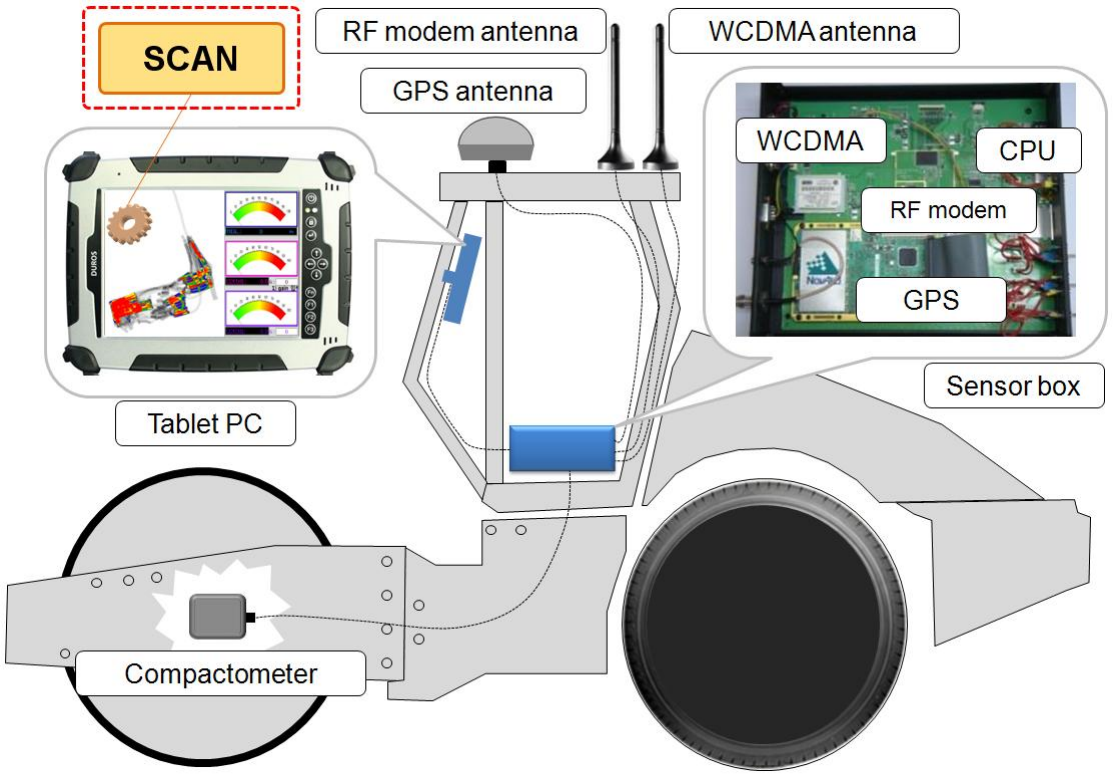
Compaction roller with sensors and instruments for IC.

Many sensors are mounted on a roller to gather data in real time for the purpose of location-based soil compaction analysis. Figure 2 shows the sensors and other equipment mounted on the roller. The main sensor is GPS for positioning and the compactometer for gathering soil compaction data.

The compactometer is a well-known device that measures the drum’s movement and processes the resulting signals to provide continuous relative values of a material’s level of compaction stiffness [13]. The device outputs the following three values: compaction meter value (CMV) for evaluation of the degree of compaction, resonant meter value (RMV), and vibrating frequency. This process is known in Europe as continuous compaction control (CCC), and is widely used. This technique has also been combined with an integrated GPS to provide a complete GIS-based record of the earthwork site to improve infrastructure performance, to reduce costs and construction duration, and to improve safety [4]. In the United States, the Federal Highway Administration [14] and State Department of Transportation (DOT) have also launched a new study, “*Accelerated Implementation of Intelligent Compaction Technology for Embankment Subgrade Soils, Aggregate Base, and Asphalt Pavement Materials (DTFH61-07-C-R0032)*” to accelerate the understanding and implementation of intelligent and continuous compaction control technology.

Two types of wireless communication modems are used; one is used for sending gathered data to the monitoring center and the other is used for receiving correction messages from a base GPS station. The latter is used only when RTK-GPS positioning is used, and it can be neglected when using SBAS or SPP positioning. In this study, the focus was on the cases where SBAS and SPP positioning are used with a low cost GPS receiver.

It is necessary to display compaction states of ground to the driver for controlling compaction work. For this purpose, a tablet PC is mounted on the front of the driver’s seat, and it displays the compaction state of the ground on which the rollers are moving in real time. SCAN works in application software installed on the tablet PC. It processes data gathered by the GPS and compactometer and shows the results to the driver and guides the driving. To perform this real time guiding and controlling work, SCAN requires capability for fast data processing and accurate analysis of the location-based soil compaction data.

## 3. Main Process of SCAN to Evaluate Soil Compaction States

**Figure 3.**
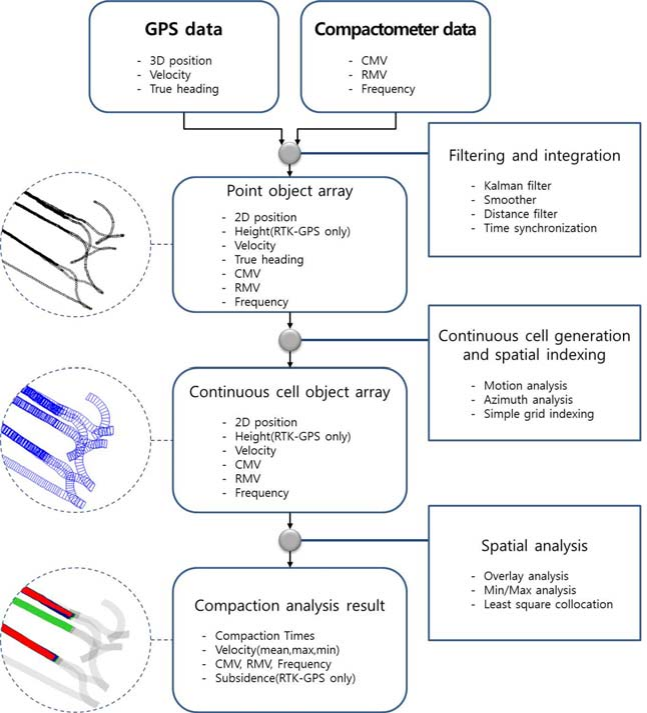
Main process of SCAN.

SCAN’s process of primary data processing and algorithms configuring each step are shown in Figure 3. The purpose of this process is to analyze data from multiple rollers quickly and to minimize the error range of the low cost GPS receiver.

The main process includes three steps: (1) generating point-type spatial objects by converting and integrating data from GPS and the compactometer mounted on a roller; (2) converting point-type data to continuous quadrangle cell-type spatial objects and building a spatial index; and (3) producing compaction results through statistical and spatial analysis.

In Step (1), GPS coordinates are filtered by three filters, GPS velocity based on the Kalman Filter (GVKF), the smoother, and the distance filters, to reduce errors on GPS coordinates and to ensure ease of generating a continuous quadrangle cell. The height data of the GPS coordinates are often used when using RTK-GPS positioning, but they can be neglected when using other positioning methods (SBAS positioning or SPP) because the accuracy of the height coordinates in such methods is low. Then, the filtered GPS data and compactometer data are integrated on the basis of time to be converted into point-type spatial objects. In Step (2), using properties of point objects, 2-D coordinates, grid azimuth and time, the movement of a roller is analyzed and continuous quadrangle cells are generated. These cells can successfully model the roller’s continuous path of movement and includes every datum gathered by the GPS and compactomter to be adequate for soil compaction analysis. Finally, spatial indexes are generated for cells for fast data finding and analyzing. In Step (3), some spatial analysis is performed using cell objects to obtain analysis results for investigating compaction states of the ground. Then, the final result with the analysis result of the whole area is calculated by least square collocation.

## 4. Data Processing Methods of SCAN

Several data processing methods of SCAN can be divided into the following three categories: filtering, spatial object modeling and spatial analysis.

### 4.1. Filtering

Compaction properties such as soil stiffness, the number of roller passes, vibration, *etc*. obtained during roller operation are strongly related to GPS coordinates. Therefore, it is very important to obtain more accurate and reliable GPS coordinates in the IC. Accuracy of the GPS positioning result is significantly affected by the reception environment of the GPS signal. The GPS signal may be frequently blocked or impaired by natural or artificial obstacles, and multipath errors can arise in GPS measurement. Therefore, adequate estimation filters to GPS coordinates are required for improving positional accuracy and detecting gross errors, especially in the case of using a low cost GPS receiver.

In this study, three types of filters were used to remove gross errors and to obtain correct GPS coordinates. First, GVKF was used to improve the GPS positioning results using the velocity and true azimuth calculated by the Doppler phenomena of the GPS carrier. Second, a least squares smoother filter with first-order to third-order polynomials was used to remove residual gross errors occurring whenever the positioning data deviated from a smoothed polynomial line generated from adjacent GPS positions. Third, a distance filter was employed to set the minimum distance and create spatial objects with quadrangle cells more effectively. Table 1 shows the filters used to manage GPS coordinates in SCAN and their related values.

Among the filters listed in Table 1, GVKF has the most significant effect on the quality of the GPS coordinates to improve accuracy and remove gross errors. Its effect appears more clearly in the case of a poor GPS signal reception environment. The reason for using this filter is to utilize the low cost GPS receiver for the IC work. The content and structure of the GVKF are as follows.

A KF uses a state Equation (1) and an observation Equation (2) to model the state of a moving vehicle in terms of linear dynamics [15]:

(1) $$X_{k+1}={\mathrm{AX}}_{k}+{\mathrm{BU}}_{k}+\omega_{k}$$

(2) $$Y_{k+1}={\mathrm{HX}}_{k+1}+z_{k+1}$$

where *X_k_* is a state vector, *U_k_* is a control vector, and *Y_k_* is a measurement vector at time k. *A* is a transition matrix and relates the state vector at time *k* to the state vector at time *k* +1. Matrix *B* relates the control vector to the state vector. Matrix *H* in the observation equation relates the state vector to the measurement vector. *ω_k_* is a process error, and *z_k_* represents a measurement error and they are assumed to be independent of each other and with normal probability distributions like below:

(3) p(ω)∼N(0,Q)

(4) p(z)∼N(0,R)

Equations (1) and (2) of the general KF can also be transformed into Equations (5) and (6) of GVKF. The three-dimensional (3-D) movements of a roller with a north coordinate (*N_k_*), an east coordinate (*E_k_*), and a height coordinate (*H_k_*) as state variables are modeled with these two equations. In Equation (5), the coordinate and state vectors at time *k* +1 are predicted with the coordinate, velocity, and control vectors at time *k*: north velocity (*V_N_k__*), east velocity (*V_E_k__*), and height velocity (*V_H_k__*). It is possible because the product of the time difference (*dt*) and the velocity is the position difference between time *k* and *k*+1. Thus, the process error matrix (*ω_k_*) can consist of the product of the time difference and the errors of the velocity at time *k* because the error in the measured velocity causes a positional error:

(5) $$\left[\begin{array}{l} N_{k+1}\\ E_{k+1}\\ H_{k+1} \end{array}\right]=I_{3\times 3}\;\left[\begin{array}{l} N_{k}\\ E_{k}\\ H_{k} \end{array}\right]+\left[\begin{array}{ccc} \mathrm{dt} & 0 & 0\\ 0 & \mathrm{dt} & 0\\ 0 & 0 & \mathrm{dt} \end{array}\right]\;\left[\begin{array}{l} V_{N_{k}}\\ V_{E_{k}}\\ V_{H_{k}} \end{array}\right]+\omega_{k}$$

(6) $$Y_{k+1}=I_{3\times 3}\;\left[\begin{array}{l} N_{k+1}\\ E_{k+1}\\ H_{k+1} \end{array}\right]+z_{k+1}$$

The data used for estimating positions can be obtained from the messages transmitted from a GPS receiver. In general, both 3-D coordinates and height velocity are included in NMEA messages or other receiver manufacturer’s specific messages. The north and east velocities should be calculated using the horizontal velocity (*V_HOR_*) and the true azimuth (*ψ_T_*) from GPS messages. First, the grid azimuth (*ψ_G_*) is calculated by applying meridian convergence to the true azimuth. Next, the north velocity [*V_HOR_*×cos(*ψ_G_*)] and the east velocity [*V_HOR_*×sin(*ψ_G_*)] can be calculated from the horizontal velocity and the grid azimuth.

The estimation process for obtaining the positions of the improved accuracy using the gathered data and the coefficient matrix in Equations (5) and (6) is as shown below.

First, in the initialization step, the initial values of the state vector and the covariance matrix of the process error at the initial time are determined. Next, in the prediction step, the state vector ( ${\hat{X}}_{k+1}^{-}$) and its covariance matrix ( $P_{k+1}^{-}$) at time *k*+1 are predicted using Equations (7) and (8), respectively:

(7) $${\hat{X}}_{k+1}^{-}={A\hat{X}}_{k}+{\mathrm{BU}}_{k}$$

(8) $$P_{k+1}^{-}={\mathrm{AP}}_{k}\;A^{T}+Q_{k}$$

Next, in the correction step, the predicted state vector and its covariance matrix are corrected using the measurement result at time *k*+1. In this step, the value of the Kalman gain (*K*_*k*+1_) is determined using Equation (9). Then the corrected state vector (*X̂*_*k*+1_) and its covariance matrix (*P*_*k*+1_) are calculated using Equations (10) and (11):

(9) $$K_{k+1}=P_{k+1}^{-}H^{T}{\left({\mathrm{HP}}_{k+1}^{-}H^{T}+R_{k+1}\right)}^{-1}$$

(10) $${\hat{X}}_{k+1}={\hat{X}}_{k+1}^{-}+K_{k+1}(Y_{k+1}-{H\hat{X}}_{k+1}^{-})$$

(11) $$P_{k+1}=(I-K_{k+1}H)P_{k+1}^{-}$$

Finally, the prediction and correction steps should be repeated at every data collection time to estimate the coordinates over the entire time span with an improved accuracy.

The value of most matrix in Equations (7–11) except *Q_k_* and *R_k_* are defined in Equation (5) and (6). It is important to define the value of *Q_k_* and *R_k_*, the Variance-Covariance Matrix (VCM) of the process and measurement error, exactly because the suitability of their values affects a lot on the accuracy of the estimation. It would be ideal to use a GPS receiver that outputs the VCM of the process and measurement error. However, such devices are rare and the most GPS receiver only output the RMSE of coordinates. So, in this study the empirical methods were applied to model the VCMs. We collected GPS data for over six hours at fixed position by the type of positioning methods, SPP, SBAS and RTK, and determined the error of 3-D velocity and 3-D coordinates, which used for determining the value of *Q_k_* and *R_k_*. *Q_k_* was set to a constant and all the elements of it were determined empirically because it is hard to determine the value of it every time of data collection. In case of *R_k_*, only covariance between coordinates were determined empirically because diagonal elements of it can be determined using RMSE of coordinates gathered from the output messages of GPS receiver at every measurement time:

(12) $$\omega =\mathrm{dt}\left[\begin{array}{l} \sigma_{V_{N}}\\ \sigma_{V_{E}}\\ \sigma_{V_{H}} \end{array}\right],\;\;\;\;\;Q=E(\omega \cdot \omega^{T})={\mathrm{dt}}^{2}\left[\begin{array}{ccc} \sigma_{V_{N}}^{2} & \sigma_{V_{N}\;V_{E}} & \sigma_{V_{N}\;V_{H}}\\ \sigma_{V_{E}\;V_{N}} & \sigma_{V_{E}}^{2} & \sigma_{V_{E}\;V_{H}}\\ \sigma_{V_{H}\;V_{N}} & \sigma_{V_{H}\;V_{E}} & \sigma_{V_{H}}^{2} \end{array}\right]$$

(13) $$z_{k}=\left[\begin{array}{l} \sigma_{N_{k}}\\ \sigma_{E_{k}}\\ \sigma_{H_{k}} \end{array}\right],\;\;\;\;\;R_{k}=E(z_{k}\cdot z_{k}^{T})=\left[\begin{array}{ccc} \sigma_{N_{k}}^{2} & \sigma_{\mathrm{NE}} & \sigma_{\mathrm{NH}}\\ \sigma_{\mathrm{EN}} & \sigma_{E_{k}}^{2} & \sigma_{\mathrm{EH}}\\ \sigma_{\mathrm{HN}} & \sigma_{\mathrm{HE}} & \sigma_{H_{k}}^{2} \end{array}\right]$$

The process error vector and its VCM are described in Equation (12), and the values of the elements of VCM (*Q*) determined empirically by the positioning method type are listed in Table 2. On the other hand, the measurement error vector and its VCM are described in Equation (13), and the values of the non-diagonal elements of VCM (*R_k_*) determined empirically by positioning method type are listed in Table 3.

It can be seen in Table 2 that the variances of the 3-D velocity are very small even though the accuracy of the positioning method (e.g., SPP) is very low. Therefore, the estimation process of GVKF, which uses the 3-D velocity as a control vector, can greatly improve the accuracy of the 3-D coordinates. Moreover, the effect of GVKF greatly increases when GPS positioning methods with low accuracy are used. In this study, the performance of GVKF was verified through a field test, and the test results are presented in Section 5.2.

### 4.2. Spatial Object Modeling

**Figure 4.**
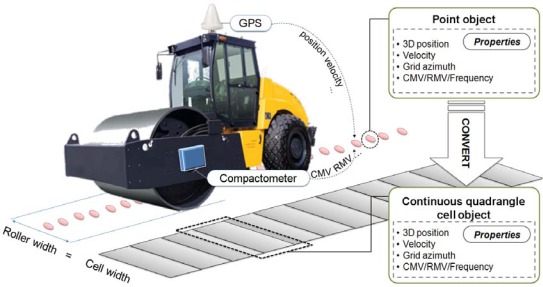
Spatial object models of SCAN.

Two types of spatial object models were used for modeling the vibrating roller’s continuous trajectory and analyzing compaction states of ground. The filtered data converted into point objects include the following properties: 3-D position, velocity obtained from GPS, and compaction properties such as degree of compaction, CMV, RMV, and vibration frequency of the roller. Next, the point objects were converted to continuous quadrangle cell objects having all properties of the point objects. Figure 4 shows the converting and creating processes of the spatial object models.

**Figure 5.**
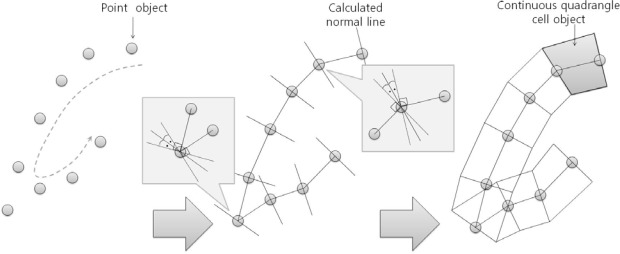
Algorithm for converting point objects to continuous quadrangle cell objects.

Figure 5 shows the algorithm of converting point objects to cell objects using two steps. First, the azimuth angles perpendicular to the connect lines between the first and second point and between the second and third point were calculated. Next, the average of the two azimuths to determine the azimuth of the second point was configured. This process was iterated to calculate each azimuth for every point.

Lastly, the two points that were located at a half-width of the roller drum from the center point were calculated. Then, continuous quadrangle cell objects were created by connecting these points with the adjacent points. The properties of each cell were calculated by averaging the properties of the adjacent two points.

However, this algorithm had a serious weak point in that the quadrangle cell cannot be created when the compaction roller changes its direction sharply because the normal lines of the two points cross each other. One of the solutions to this problem was the elimination of adjacent points until a quadrangle cell is formed. Therefore, this solution was applied to compensate the shortcoming in the generation of continuous quadrangle cell objects.

**Figure 6.**
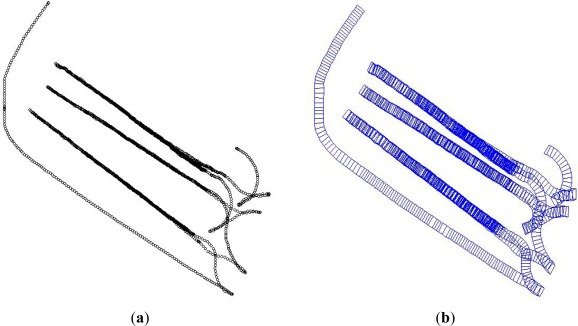
Example of converting point objects to continuous quadrangle cell objects. (**a**) Point objects data; (**b**) Continuous quadrangle cell objects were generated by spatial object modeling methods.

Figure 6 shows the result of generating continuous quadrangle cell objects (Figure 6(b)) from point objects (Figure 6(a)) by the spatial object modeling methods suggested in this study. In this figure, the quadrangle cell objects that describe the roller’s actual continuous motion were successfully generated.

### 4.3. Spatial Analysis

GPS and compaction sensors mounted on a vibrating roller can produce at least 3,600 positions and compaction relating data per hour. To treat this quantity of data quickly and effectively, an appropriate spatial index and processing method was required for an analysis.

In this study, a simple grid index, which is frequently used for spatial indexing of earth observation data, was employed to realize continuous cell objects widely distributed over a large area [16]. Overlay analysis, the general method used in the GIS analysis, was also employed in SCAN.

**Figure 7.**
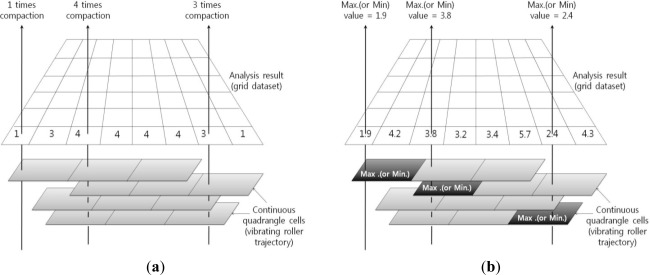
Overlay analysis method. (**a**) Compaction times analysis; (**b**) CMV/RMV/Frequency analysis.

Figure 7 shows the concept of the overlay analysis method developed in this study. The first step involved making a grid type data set covering the entire analyzing domain, and to search vertically-superposed cells on each grid position. A number of superposed cells represent a number of vibratory roller passes, and minimum or maximum values of the compaction properties in overlaid cells can be chosen for analyzing the compaction states of the ground.

The Least Squares Collocation (LSC) method was also used to produce reasonable compaction results for the entire earthwork area by considering the correlation among the adjacent values to even out the data distribution, to interpolate the data for the areas with no data, and to prevent gross errors. Typically, the general Kriging method and many modified Kriging methods have been used at the geo-statistical areas. Although the LSC and Kriging methods are not exactly the same [17], the differences between them are not significant [18].

## 5. Verification of SACN throughout Field Tests

SCAN was evaluated from the processing sample data and test operation of the monitoring software in terms of accuracy of soil compaction analysis, capability of processing low cost GPS data, and processing speed to manage multiple roller data in real time.

### 5.1. Accuracy Tests of Soil Compaction Analysis

**Figure 8.**
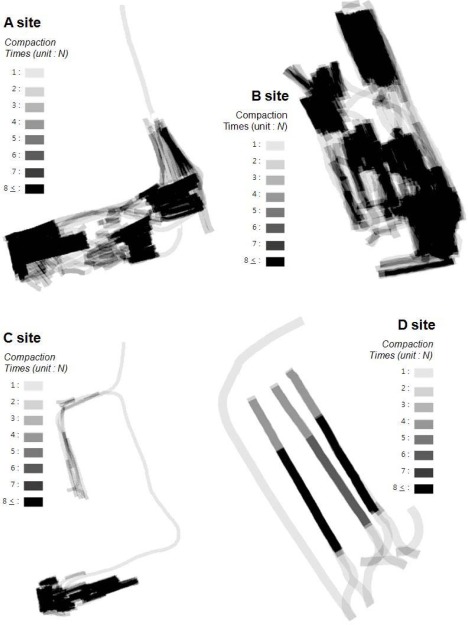
Analysis results of compaction times from GPS data only (at sites A to D).

Sample data gathered from five different sites (A, B, C, D, E) were processed to verify the accuracy of analysis from SCAN. Figure 8 shows the analysis results of the number of roller passes (compaction times) from four sites (A to D). A path with more than eight roller passes is displayed with a very dark color. Paths with less than seven roller passes are shown with lighter colors.

The results show that the roller’s continuous motions were modeled very accurately from the continuous quadrangle cells, and the compaction times were analyzed accurately by overlay analysis methods. This analysis makes it possible to check if the field work was performed according to regulations.

**Figure 9.**
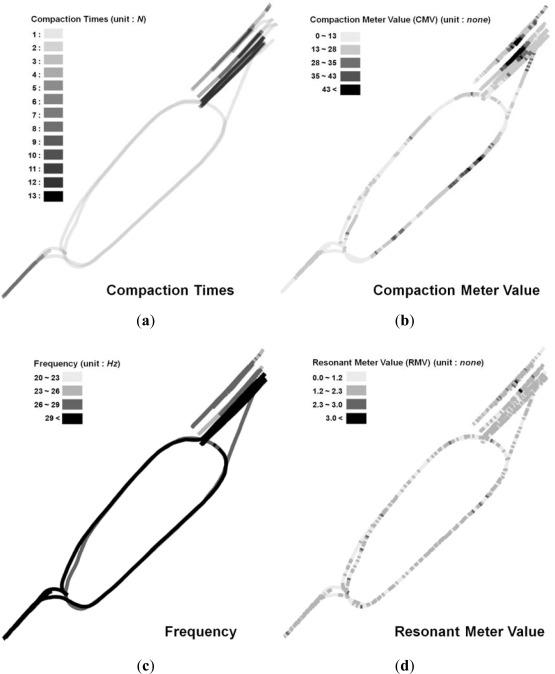
Field data analysis results at site E. (**a**) Compaction times from GPS data only; (**b**) Compaction meter value (CMV); (**c**) Vibrating frequency; (**d**) Resonant meter value (RMV). The values of (**b**), (**c**) and (**d**) were obtained from the combination of GPS and compactometer data.

Figure 9 shows analysis results of the number of roller passes, compaction properties, CMV, RMV, and vibration frequency using data from a roller at site E. The roller was operated at 20, 26, and 30 Hz, and there was a maximum of twelve roller passes at site E. Especially, Figure 9(b) shows the CMV and relative degree of compaction obtained from the compactometer. The CMV of the area through which the roller had passed at least four times was generally greater than the CMV of the other areas. Thus, the number of passes, the vibration frequency, the CMV varying from 0 to 80, and the RMV varying from 0 to 4 were effectively displayed by SCAN in order to characterize the state of roller compaction at site E.

### 5.2. Utilization Tests of Low Cost GPS Receiver

**Figure 10.**
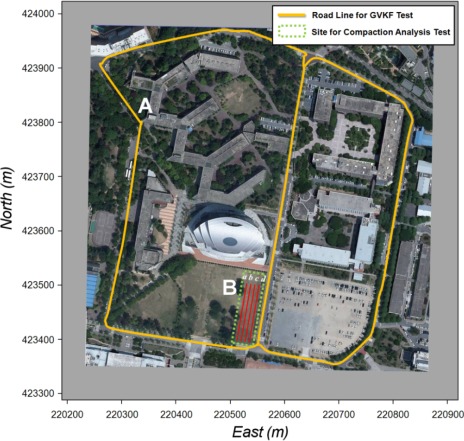
Test sites for utilization test for GVKF and SCAN’s capability. Road line (site A) for GVKF capability to a low cost GPS receiver mounted on a moving vehicle. A compaction site (site B) for SCAN capability to a low cost GPS receiver mounted on a roller under similar environments of compaction work.

GVKF and SCAN’s capability was tested and evaluated for its improvement of positioning accuracy and analysis of compaction properties obtained by a roller trajectory modeling with a low cost GPS receiver. For this evaluation, two tests were performed: (1) a utilization test for GVKF capability to improve positioning accuracy of the result provided by the low cost GPS receiver used by the SPP and SBAS signals mounted on a moving vehicle, and (2) a utilization test for SCAN (with GVKF), analyzing the roller trajectory and compaction properties from the roller’s low cost GPS results which result in precise compaction control work of the IC under similar compaction work environments. Figure 10 shows the selected test sites for the utilization tests of GVKF (site A) and SCAN capability (site B), respectively.

**Figure 11.**
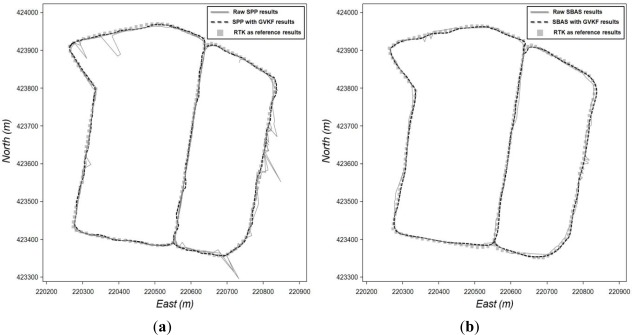
Effects of GVKF for improving positioning accuracy of low cost GPS results with respect to the moving vehicle. (**a**) Effect of GVKF for the SPP results; (**b**) Effects of GVKF for SBAS results.

The first step of the utilization test of GVKF was surveying the center of the selected road line (site A in Figure 10) by RTK positioning and using the RTK results as reference values for accuracy evaluation of low cost GPS results and those with GVKF respectively. Then, we drove the vehicle across the road in a variety of GPS reception environments and obtained SBAS and SPP results with respect to the center of the road line using the low cost GPS receivers. The raw SPP, raw SBAS results and those with GVKF results will be compared with the RTK results in order to evaluate GVKF capability to improve the accuracy of the low cost GPS positioning. Figure 11(a) shows the raw and GVKF applied results from SPP positioning and Figure 11(b) shows those from SBAS positioning based on the RTK results.

**Figure 12.**
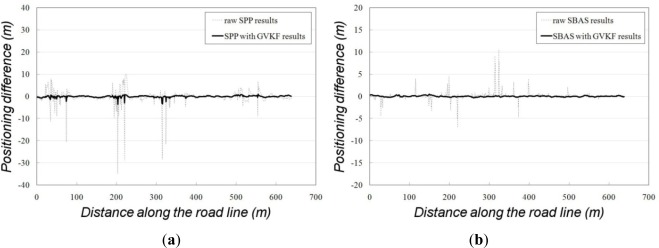
Comparison of positioning accuracy based on RTK results. (**a**) Positioning differences between RTK and raw SPP results (dashed line), RTK and SPP with GVKF results (full line); (**b**) Positioning differences between RTK and raw SBAS results (dashed line), RTK and SBAS with GVKF results (full line).

As shown in Figure 11(a,b), there were many gross errors in the raw SBAS and SPP results (full lines) in which many positions were placed outside of the road. After being filtered by GVKF, most positions were placed inside the road, so it was logical that the accuracy of filtered results was improved considerably by GVKF. In order to analyze the improvement of accuracy more clearly, we compared and summarized all results (raw, GVKF and RTK) according to the positioning methods as shown in Figure 12 and Table 4.

Figure 12(a) shows the positioning differences between the raw SPP (dashed line) and RTK results, SPP with GVKF (full line) and RTK results. Figure 12(b) also shows the positioning differences between the raw SBAS (dashed line) and RTK results, SBAS with GVKF (full line) and RTK results. In Figure 12 and Table 3, there are many gross errors exceeding tens of meters included in the raw SPP and SBAS results and these gross errors could be reduced considerably by GVKF. These situations indicate that GVFK has sufficient capability to improve the accuracy of SPP, and SBAS positioning could be used for the compaction control work of IC.

In the utilization test of SCAN capability, two types of GPS receivers were installed on the compaction roller: one for SBAS and SPP positioning, and the other for RTK positioning. Then, the roller was driven along four paths (site B in Figure 10) under similar compaction work environments and the positioning results were obtained from the three types of GPS receivers. To analyze the positioning accuracy improvement by GVKF, the filtered results of SPP and SBAS methods are compared with the RTK results presented in Table 5.

Table 5 shows the accuracy assessment result for the GVKF-filtered SBAS and SPP results. It is seen that the overall positioning accuracy is better than that for results of the previous road line test because the GPS signal reception environment is good. In addition, the effect of GVKF is slightly reduced in this test because the moving speed of the compaction roller is lower than that of the vehicle.

**Figure 13.**
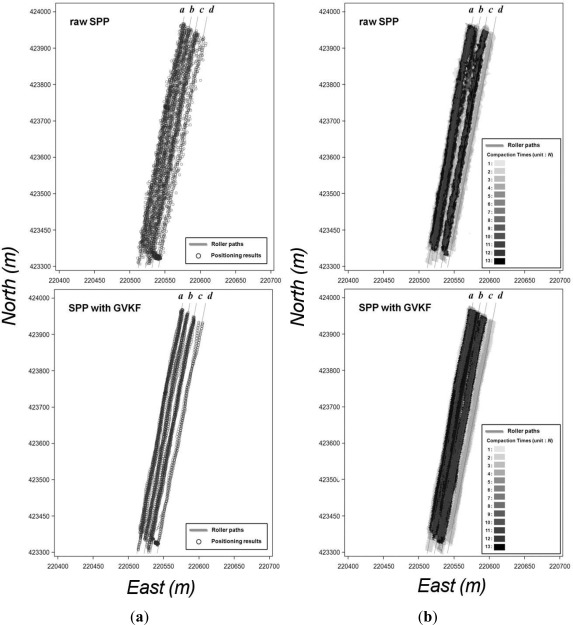
Utilization test results of SCAN capability for precise compaction analysis using SPP. (**a**) Point positions from raw SPP (upper) and SPP with GVKF (lower) method; (**b**) Compaction times analysis by SCAN on each of the positioning methods.

**Figure 14.**
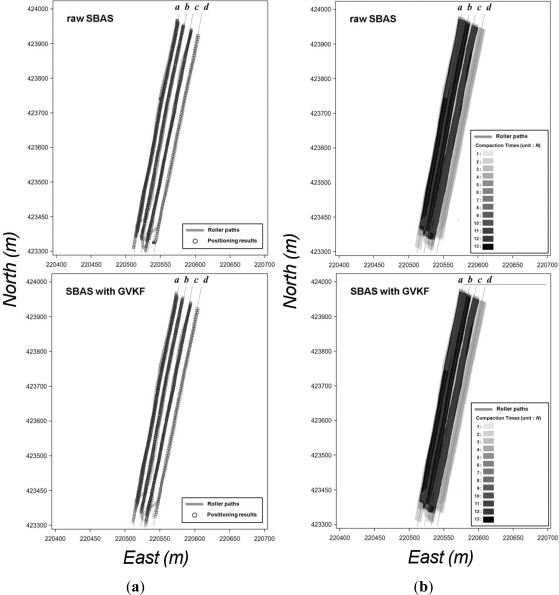
Utilization test results of SCAN capability for precise compaction analysis using SBAS. (**a**) Point positions from raw SBAS (upper) and SBAS with GVKF (lower) method; (**b**) Compaction times analysis by SCAN on each of the positioning methods.

Finally, each result of compaction analysis was compared using three types of GPS positioning (SPP, SBAS and RTK) together with the GVKF-filtered SPP and SBAS results in order to evaluate SCAN capability for precise compaction analysis. Figures 13 and 14 show the results of assessment for improving the possibility of the SPP and SBAS positioning result's usage, respectively, at the precise compaction control work.

Figure 13(a) shows the raw SPP and SPP with GVKF positioning results plotted in the form of points and Figure 13(b) shows the trajectory width of the compaction roller modeled by each positioning result according to the roller’s path. Figure 14(a,b) also represent the same content for each of the raw SBAS and SBAS with GVKF positioning methods to that of Figure 14(a,b), respectively.

It was found in Table 4, and in Figures 13(b) and 14(b) that compaction analysis results generated from raw SPP data are inadequate for both simple IC work such as compaction times monitoring and precise compaction control work of IC. However, it is shown that the compaction analysis results generated from GVKF-filtered SPP results could be used for compaction times monitoring.

Conversely, the compaction analysis results generated from all SBAS results (raw and GVKF-filtered) were very similar to those from the RTK results. Therefore, it was possible to use the raw SBAS results (without GVKF) directly for both simple and precise compaction control work of IC. However, the accuracy of unfiltered SBAS positioning results can be degraded in a poor GPS signal reception environment, and gross error in positioning results can seriously affect the compaction analysis. Therefore, using GVKF-filtered SBAS results are more stable for precise compaction analysis.

From the above results of utilization tests, it is apparent that SCAN including GVKF can be used effectively for simple or precise compaction control work of IC using the positioning data obtained only from a low cost GPS receiver, SPP and SBAS.

### 5.3. Processing Capability Tests of Real Time Compaction Monitoring

The processing capability of compaction monitoring software which uses SCAN as a soil compaction analysis module was tested. Four rollers were monitored via the WCDMA network, and their data were gathered from GPS and a compactometer that were sent to the monitoring software in real time.

**Figure 15.**
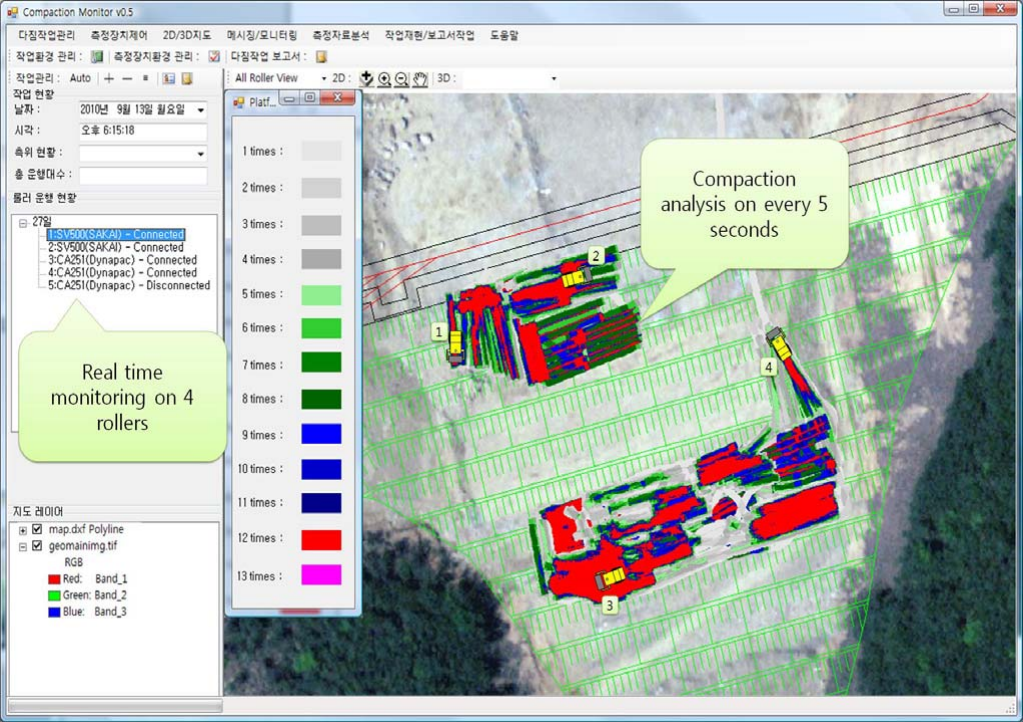
Screen capture of real time compaction monitoring software.

Figure 15 shows a screen capture of the monitoring software. In the figure, four rollers are sending data at one-second intervals, moreover the monitoring software analyzes the data and displays the analysis results every five second. The test results show that SCAN is adequate for real-time analysis for soil compaction monitoring using data from multiple rollers.

## 6. Conclusions

In this study, a software module, SCAN, was developed as an effective analysis tool to evaluate the compaction states of the ground for the IC work. A process composed of many methods was developed for very accurate analysis and utilization of a low cost GPS and of SCAN with GVKF. From the results of this study, the following conclusions are drawn:

- A spatial object based on a continuous quadrangle cell is very effective in modeling the trajectory of rollers and compaction properties during compaction. Modeling spatial objects with appropriate filters and topology structures makes the adoption of various GIS analyzing methods more convenient.
- It is possible to utilize low cost GPS for the IC work by applying filters to improve positional accuracy and to remove gross errors. Especially, GVKF, a main filter, works well to improve the GPS positioning results without any external sensors. By using filters included in SCAN, the SPP results can be used for simple IC work such as compaction time analysis and SBAS results can be used for IC work that requires a higher level of precision.
- Overlay analysis and spatial indexing based on GIS analysis is more effective since the data of continuous quadrangle cells are adequate to display the state of roller compaction in the field. These analysis method and structures are able to process data from multiple rollers in real time because their processing speed is sufficiently fast to support the real time compaction state analysis work.
- For further study, SCAN will be improved so that it can be used in the system that automatically controls compaction rollers for more accurate compaction work. This is important because one of the ultimate purposes of the IC system is an automatic machine control. For this, a new spatial indexing method adequate for the continuous cell object arrays will be developed to improve the processing capability.

## Figures and Tables

**Table 1.** Filters used for improving GPS positioning results.

**Order**	**Applied filter**	**Used data**	**Function**
1	GVKF (GPS velocity based kalman filter)	3-D coordinates, covariance matrix, time, velocity, grid azimuth	Accuracy improvement and gross error detection
2	Smoother	3-D coordinates	Residual gross error detection and smoothing the trajectory
3	Distance filter	3-D coordinates, time	Maintaining minimum distance between adjacent two points for effective spatial modeling of roller trajectory

**Table 2.** Empirically determined values of VCM (*Q*)’s elements by positioning method type.

**Positioning Methods**	**Elements of VCM(***Q***) (unit: m^2^/s^2^)**					
	$\sigma_{V_{N}}^{2}$	$\sigma_{V_{E}}^{2}$	$\sigma_{V_{H}}^{2}$	*σ_V_N_V_E__*(=*σ_V_E_V_N__*)	*σ_V_N_V_H__*(=*σ_V_H_V_N__*)	*σ_V_E_V_H__*(=*σ_V_H_V_E__*)
SPP	0.00031	0.00045	0.00145	−3.90E−06	9.76E−05	−4.51E−05
SBAS	0.00024	0.00041	0.00125	−2.31E−05	3.16E−05	−1.32E−04
RTK	0.00016	0.00021	0.00127	−1.12E−06	3.62E−05	1.23E−05

**Table 3.** Empirically determined values of VCM (*R_k_*)’s non-diagonal elements by positioning method type.

**Positioning Methods**	**Elements of VCM(***R_k_***) (unit: m^2^)**		
	*σ_NE_*(=*σ_EN_*)	*σ_NH_*(=*σ_HN_*)	*σ_EH_*(=*σ_HE_*)
SPP	0.6601	0.4156	−0.7703
SBAS	0.0518	0.2823	0.4499
RTK	−7.10E−07	−1.10E−06	1.07E−05

**Table 4.** Statistics of positioning differences of various positioning methods based on RTK results in test road line (site A in Figure 10).

**Positioning Methods**	**raw SPP (unit: m)**	**SPP with GVKF (unit: m)**	**raw SBAS (unit: m)**	**SBAS with GVKF (unit: m)**
MIN	−34.771	−3.551	−6.893	−0.325
MAX	10.075	0.983	10.529	0.505
MEAN	−0.179	−0.067	0.078	0.007
Std. dev.	3.360	1.253	0.844	0.127

**Table 5.** Statistics of positioning differences of various positioning methods based on RTK results in test compaction site (site B in Figure 10).

**Positioning Methods**	**raw SPP (unit: m)**	**SPP with GVKF (unit: m)**	**raw SBAS (unit: m)**	**SBAS with GVKF (unit: m)**
MIN	−1.611	−0.690	−0.346	−0.325
MAX	1.668	0.856	0.424	0.505
MEAN	−0.009	−0.009	−0.011	0.003
Std. dev.	0.487	0.255	0.127	0.117
